# Natural versus artificial light exposure on delirium incidence in ARDS patients

**DOI:** 10.1186/s13613-020-0630-8

**Published:** 2020-02-05

**Authors:** Amir Vahedian-Azimi, Farshid R. Bashar, Abbas M. Khan, Andrew C. Miller

**Affiliations:** 1 Trauma Research Center, Nursing Faculty, Baqiyatallah University of Medical Sciences, Tehran, Iran; 2 Anesthesia and Critical Care Department, Hamadan University of Medical Sciences, Hamadan, Iran; 3 The MORZAK Collaborative, Greenville, NC USA; 4 Department of Emergency Medicine, East Carolina University Brody School of Medicine, 600 Moye Blvd, Mailstop 625, Greenville, NC 27834 USA

**Keywords:** Natural light, Delirium, Intensive care unit, ARDS

We read with interest the study by Smonig et al. on the impact of natural light (NL) exposure on delirium-associated outcomes in mechanically ventilated (MV) intensive care unit (ICU) patients [1]. In this single-center, prospective, observational study, the authors report an improvement in the secondary outcomes of hallucination incidence and haloperidol administration for agitation. No difference in delirium incidence or duration, MV duration, self-extubation, ICU or hospital length-of-stay (LOS), or mortality was observed [1]. We request clarification on whether the cumulative doses of haloperidol differed.

Smonig’s findings differ from our observations. We have conducted a longitudinal cohort study of 16,000 ICU patients with acute respiratory distress syndrome (ARDS) on MV from 21 ICUs (10 mixed, 5 surgical, 6 medical) from 6 academic medical centers [2, 3]. Here, we report the results of a retrospective secondary analysis of 4200 patients from the mixed medical–surgical ICUs of two academic hospitals to assess the impact of NL exposure on delirium incidence. Each ICU had the same layout including 10 beds; 5 with adjacent windows allowing for NL (circadian pattern), and 5 positioned 13 m from the nearest window (artificial light: AL). Delirium was defined according to the DSM-IV-TR [4], and was assessed three times daily by the bedside nurse and researcher (kappa agreement coefficient 0.801–0.902) using the Confusion Assessment Method for the ICU (CAM-ICU) [5]. We performed both unadjusted and adjusted logistic regression accounting for: year, diagnosis, age, sex, vital signs, illness severity (APACHE-II score), development of ventilator-associated pneumonia, microbiology results, presence of an multiple drug resistant pathogens, MV duration, LOS (ICU, hospital), and survival. We found that AL patients had a 2.35- and 2.39-times greater incidence of delirium by unadjusted and adjusted logistic regression, respectively.

Methodological differences in delirium definition, screening method and frequency, criteria for NL group, and population studied may contribute to the outcome heterogeneity across studies (Table 1) [1, 6–9]. Six studies utilized a validated delirium screening tool (Table 1), whereas one did not [8], and one included (as a positive) any patient treated with haloperidol (regardless of screen result) [6]. Furthermore, two studies required a positive delirium screen on ≥ 2 consecutive days to be classified as delirium [1, 7]. Moreover, the light exposure definitions vary considerably across studies. Three studies compare patients in rooms with or without windows [1, 7, 8], whereas in two studies, all patients have NL exposure to differing degrees [6, 9]. The assessed patient populations differ as well. Whereas we found improved delirium outcomes in ARDS patients, who often have greater illness severity and longer ICU LOS than the general ICU patient population, no difference was observed in other ICU populations [1, 6–8]. Our data suggest that further investigation in defined ICU sub-populations may provide an opportunity to better identify those likely to benefit from NL exposure. Such studies should capitalize on transparency using clear and reproducible of key variables including the definitions of delirium and NL exposure. Based on the current level of evidence, it would be premature to discard a therapeutic role for NL exposure in critically ill patients.

**Table 1:** Design heterogeneity in studies on the effects of natural light exposure on patients in the intensive care unit

Author (reference)	Design (*N*)	Sample size calculation	Delirium definition	Screening tool	Screen frequency (no./day)	ICU patient population	Delirium incidence or severity with NL exposure
Our study	Retrospective analysis of prospective study (181)	Yes^a^	DSM-IV-TR	CAM-ICU	3	Long stay medical and surgical with ARDS	Decreased
Arenson [6]	Retrospective (1010)	No	Not reported	CAM-ICU	3	Post-operative	No change
Estrup [5]	Retrospective (183)	No	Not reported	CAM-ICU^b^	2	Unspecified	No change
Kohn [7]	Retrospective (6631)	No	Not reported	None^c^	1	Medical ICU patients	No change
Smonig [1]	Prospective, observational (195)	Yes^a^	Not reported	ICDSC^c^	2	On MV of any etiology/duration	No change^d^
Zaal [8]	Prospective, before–after (130)	No	Not reported	CAM-ICU	1	Medical and surgical	No change

*ARDS* acute respiratory distress syndrome, *CAM-ICU* confusion assessment method for the ICU, *DSM* Diagnostic and Statistical Manual of Mental Disorders, *ICDSC* Intensive Care Delirium Screening Checklist, *MV* mechanical ventilation

^a^To achieve a power of power 80% to detect a decrease of delirium from 80 to 60% (two-sided test, alpha = 0.05), the necessary sample size is 180 patients [1] would be necessary

^b^Delirium categorization included any patient treated with haloperidol, regardless of CAM-ICU screen

^c^Required a positive screen for at least 2 consecutive days to be considered positive

^d^Less haloperidol administration; less hallucinations

## Data Availability

All data generated or analyzed during this study are included in this article.

## Abbreviations

AL: Artificial light
ARDS: Acute respiratory distress syndrome
CAM-ICU: Confusion Assessment Method for the ICU
ICU: Intensive care unit
LOS: Length-of-stay
MV: Mechanical ventilation
NL: Natural light
